# Rheumatism

**Published:** 1880-04

**Authors:** 


**RHEUMATISM.**—Common rheumatism is a disease which affects the joints, the hinges of the body, in such a way, that the slightest motion of the ailing part gives pain. A creaking hinge is dry, and turns hard. A single drop of oil to moisten it makes a wonderful change, and it instantly moves on itself with the utmost facility. All kinds of rheumatism are an inflammation of the surface of the joints. Inflammation is heat; this heat dries their surfaces; hence, the very slightest effort at motion gives piercing pain. In a healthy condition of the parts, nature is constantly throwing out a lubricating oil, which keeps the joints in a perfectly smooth and easy-working condition. Rheumatism is almost always caused—indeed, it may be nearer the truth to say, that it is always the result of a cold dampness. A dry cold, or a warm dampness, does not induce rheumatism. A garment, wetted by perspiration or rain, or water in any other form, about a joint, and allowed to dry while the person is in a state of rest, is the most common way of causing rheumatism. A partial wetting of a garment is more apt to induce an attack than if the entire clothing were wetted; because, in the latter case, it would be certainly and speedily exchanged for dry garments. There are two very certain methods of preventing rheumatism. The very moment a garment is wetted in whole or in part, change it, or keep in motion sufficient to maintain a very slight perspiration, until the clothing is perfectly dried.

The failure to wear woolen flannel next the skin, is the most frequent cause of rheumatism; for a common muslin or linen or silk shirt of a person in a perspiration, becomes damp and cold the instant a puff of air strikes it, even in mid-summer. This is not the case when woolen flannel is worn next the skin.

The easiest, most certain, and least hurtful way of curing this troublesome affection is, first, to keep the joint affected wound around with several folds of woolen flannel; second, live entirely on the lightest kind of food, such as coarse breads, ripe fruits, berries, boiled turnips, stewed apples, and the like. If such things were eaten to the extent of keeping the system freely open, and exercise were taken, so that a slight moisture should be on the surface of the skin all the time; or if, in bed, the same thing were accomplished by hot teas and plentiful bed-clothing, a grateful relief and an ultimate cure will very certainly result in a reasonably short time. Without this soft and moist and warm condition of the skin, and an open state of the system, the disease will continue to torture for weeks and months and years.

Inflammatory rheumatism may, for all practical purposes, be regarded as an aggravated form of the common kind, extended to all the joints of the body, instead of implicating only one or two. For all kinds, time, flannel, warmth, with a light and cooling diet, are the great remedies.

